# McKillip Veterinary College

**Published:** 1894-10

**Authors:** 


### EDITORIAL.

### McKILLIP VETERINARY COLLEGE.

It is not always that we welcome the appearance of a new veterinary school in the country as a matter of joy to the veterinary profession, for it has often seemed that there were quite enough of them to supply the number of practitioners needed. But it should be a gratification to all veterinarians, when a new school is started, to see it established upon the solid basis indicated by the announcement of the McKillip Veterinary College at Chicago, Illinois. This school seems to be the first fruit of the labors of the United States Veterinary Medical Association, in the improvement of the grade of veterinary education. Its sixteen students were required to comply with the examinations recommended by the Association of Veterinary Faculties of North America, and will be obliged to take a curriculum of three years, of six months each.

We wish them every success.

